# Significance of MiRNA-34a and MiRNA-192 as a risk factor for nonalcoholic fatty liver disease

**DOI:** 10.1186/s43141-023-00467-z

**Published:** 2023-02-09

**Authors:** Halla M. Ragab, Wafaa M. Ezzat, Eman Mahmoud Hassan, Nabila Abd El Maksoud, Mie Afify, Mohamed D. E. Abd El-Maksoud, Wafaa Abd Elaziz

**Affiliations:** 1 Biochemistry Department, Biotechnology Research Institute, National Research Centre, Dokki, Giza, Egypt; 2 Internal Medicine Department, National Research Centre, Dokki, Giza, Egypt; 3 Clinical and Chemical Pathology Department, National Research Centre, Dokki, Giza, Egypt

**Keywords:** Nonalcoholic fatty liver disease (NAFLD), Gene expression, microRNAs (miRNAs), MiRNA-34a, miRNA-192

## Abstract

**Background and aims:**

NAFLD is one of the fast-growing health problems that affects up to 25% of people worldwide. Numerous miRNAs have been clarified as important regulators of liver pathophysiology, including NAFLD. Thus, we investigated the expression of the MiRNA-34a and MiRNA-192 as diagnostic markers for NAFLD.

**Patients and methods:**

Blood samples were collected from NAFLD cases and healthy controls. The expression profile of both studied miRNAs was detected via real-time PCR analysis.

**Results:**

The present study showed that both studied miRNAs were upregulated in NAFLD patients compared to controls. Interestingly, miRNA-34a and MiRNA-192 are upregulated in NAFLD patients with early fibrosis compared to controls [with a fold change of 4.02 ± 11.49 (*P* = 0.05) and 18.43 ± 47.8 (*P* = 0.017), respectively]. However, miRNA-34a is downregulated in NAFLD patients with advanced fibrosis compared to controls, with fold expression of 0.65 ± 1.17 (*P* = 0.831). The area under the receiver operating characteristics (AUROC) for miRNA-34a and miRNA-192 were 0.790 and 0.643, respectively; furthermore, the sensitivities and specificities were 76.7%, 100% for miRNA-34a and 63.3%, and 93.3% for miRNA-192 (*P* < 0.05). Additionally, MiRNA34a was positively correlated with hypertension and fasting blood sugar, and it also was negatively correlated with hemoglobin level and total leucocyte count (*P* < 0.05).

**Conclusion:**

The results obtained indicated that both studied miRNAs could potentially be used as diagnostic biomarkers for the early stage of liver fibrosis in NAFLD cases. Also, miRNA-34a was positively correlated with metabolic disorders associated with NAFLD such as hypertension and diabetes. However, their expression showed no association with advanced fibrosis. Thus, larger cohorts are necessitated to certify the utility of serum MiRNA-34a and MiRNA-192 in monitoring the deterioration of NAFLD.

## Highlights

● NAFLD is deliberated a complicated health problem owing to its growing prevalence, complex pathogenesis, and difficulty to diagnose.

● It is crucial to find an efficient noninvasive and affordable biomarker for earlier identification and staging of NAFLD.

● Ultimate role of miRNA-34a in lipid metabolism dysregulation may lead to NAFLD.

● MiRNA-34a and MiRNA-192 are broadly stated to play a role in the development of NAFLD and NASH.

## Introduction

Nonalcoholic fatty liver disease (NAFLD) is a common metabolic illness that affects 24% of adults worldwide [1]. NAFLD includes various medical entities starting from simple steatosis, identified by the presence of high content of fat in the liver exceeding 5–10% of liver weight in patients without other causes such as excessive alcohol consumption, to the progressive form, namely nonalcoholic steatohepatitis (NASH), which is known as an illness distinguished by lobular inflammation ballooning and hepatic fibrosis [2]. This condition may deteriorate into organ impairment or cirrhosis leading to hepatocellular carcinoma (HCC) [3, 4]. Precision histological diagnosis frequently relies on liver biopsy. However, because of this method’s drawbacks, such as the bleeding risk and others, noninvasive techniques are preferred and have attracted a lot of interest [5].

MicroRNAs are a subclass of small noncoding RNA molecules with a single chain that control the expression of genes and take part in protein translation.

Every aspect of cellular function, including differentiation and development, proliferation, apoptosis, and carcinogenesis, can be affected by them [6].

Recent research has established that miRNAs are abundant in the liver and regulate a wide range of liver functions [7, 8]. Dysregulation of miRNA expression may be a major pathogenic feature in a variety of liver disorders. Circulating miRNAs, which are incredibly stable and resistant to RNase-mediated destruction in body fluids, have emerged as attractive diagnostic tools.

Potentially valuable miRNA biomarkers need to be screened and identified. Additionally, the diagnostic accuracy of miRNAs in discriminating healthy individuals from those with NAFLD needs to be studied [9–14]. Recent research has shown a panel of miRNAs, involving MiRNA-34a, and miRNA-192 that are dysregulated in NAFLD.

miRNA-34a is highly expressed in type 2 diabetes mellitus patients as well as those with steatosis and NASH and in experimental models of NAFLD. Some studies have shown that miRNA-34a is over-expressed in the liver and serum of NAFLD patients [15]. Furthermore, increased serum levels of miRNA-34a are related to the severity of the disease, the level of liver enzymes, the degree of fibrosis, and inflammation activity [16–20].

Among all miRNAs identified, miRNA-192 was chosen due to its enrichment in hepatic stellate cells (qHSCs) and healthy liver tissue, the abundance of predicted targets, the loss of expression in cirrhotic patients, and the originality of miRNA-192 concerning liver fibrosis. In numerous malignancies, including gastric, breast, and colon cancer, miRNA-192 has been identified as an oncogene [21]. Pirola and his colleague spotted considerable fold differences in serum levels of miRNA-192in NASH vs. controls (4.4-fold change) [12]. Additionally, in mice fed a diet lacking in choline and folate, serum miRNA-192 levels are linked with the harshness of NAFLD specifically the liver pathomorphological changes [22].

Therefore, the current study’s objective was to identify serum MiRNA-34a and miRNA-192 expression patterns as diagnostic indicators of the severity of NAFLD and compare them to those of matched healthy controls.

Additionally, the relationship between the clinicopathological characteristics and the expression of both analyzed MiRNAs was assessed.

## Subjects and methods

The present study is a prospective study that involved 90 participants who attended outpatient clinics of the Center of Excellence, NRC, Egypt, between May 2020 and March 2022.

According to the FibroScan results and sonographic findings, the participants were divided into two groups. The patient group included 60 NAFLD patients, and the control group included 30 healthy subjects. The patient group was further divided into two groups: the first group included 28 NAFLD patients at an early stage of fibrosis (F0-F2), and the second group included 32 NAFLD patients with advanced stage of fibrosis (F3-F4).

All participants are between 18 and 60 years old. The mean age of participants was 40.08 ± 13.3 years.

### Exclusion criteria

- Patients with cirrhosis, hepatocellular carcinoma, or other confirmed malignancies, as well as those who have clinical or biochemical evidence of hepatitis B and C virus infection
- Patients with a history of heavy alcohol use, which is defined as a daily average alcohol intake of more than 20 g.

The National Research Centre Ethics Committee gave its approval to the study protocol, which was carried out in conformity with the Declaration of Helsinki. The approval number is 5132032021.

All patients were evaluated by history and clinical examination and measurement of anthropometric parameters including body mass index (BMI; kg/m^2^).

### Sample collection

A total of 10 ml venous blood were drawn from all study participants in the morning after a 12-h fast; a portion of the blood was collected on an EDTA tube for determination of routine blood pictures (CBC) The other portion is left to clot at room temperature. The serum was separated by centrifuging for 10 min at 3000 rpm. Sera were used immediately for other biochemical investigations and molecular analysis

### Hematological parameters evaluation

Using the automated hematology analyzer SF-300, the complete blood count was calculated (Sysmex Corporation, Japan).

### Biochemical parameters evaluation

Liver enzymes include aspartate aminotransferase (AST), alanine aminotransferase (ALT), bilirubin, alkaline phosphatase (ALP), gamma-glutamyl transferase (GGT), fasting blood glucose, serum albumin, triglycerides, cholesterol, HDL-C, and LDL-C according to the manufacturer’s instructions provıded wıth the kıts purchased from Spectrum Company, Cairo, Egypt.

NAFLD was identified sonographically, and FibroScan data were used to classify the disease’s severity.

### Molecular markers (miRNAs) detection

#### Total RNA (including miRNA) purification from serum including miRNAs

RNA was extracted immediately after sample collection from serum using miRNeasy Serum/Plasma kit (QIAGEN, Germany). Mature miRNAs were reversibly transcribed using a miRCURY LNA RT kit (QIAGEN, Germany). The cDNA product of the reverse transcription was used on the same day for relative miRNA expression of the candidate markers (MiRNA-34a, MiRNA-192) using miScript SYBR Green PCR Kit (QIAGEN, Germany). The miRNA abundance of MiRNA-34a and MiRNA-192 was normalized to that of miR-16 and was calculated based on the comparative 2^−ΔΔCt^ method

The cDNA was diluted 1:60 vıa addition of 590 μL nuclease-free water to the 10 μL RT reaction instantly before use.

Each qPCR reaction was prepared as follows, mixed by vortexing and spun down

**Table:** 

Component	Quantity
**2× miRCURY SYBR Green Master Mix**	10 μL
**cDNA template**	6 μL (diluted 1:60)
**Resuspended PCR primer mix**	2 μL
**Nuclease-free water**	2 μL
**Total reaction volume**	20 μL

Real-time PCR amplification was performed according to the following cycling parameters:

**Table:** 

Steps	Temperature	Time	Cycles
**PCR initial heat activation**	95 °C	2 min	1 cycle
**Denaturation**	95 °C	10 s	40 cycles
**Combined annealing/extension**	56 °C	60 s	
**Melting curve analysis**	60–95 °C		

### Statistical analysis

SPSS version 16.0 (Chicago, IL, USA) was used for statistical analysis with a two-sided significant criterion at *P* < 0.05. The clinical data were expressed as mean ± SD (continuous, normally distributed variables). Categorical data were summarized as percentages. The two-tailed Student’s *t*-test was used to determine the significance between the two groups. Also, qualitative variables were assessed by chi-squared (*χ*^2^) test. Correlations between both studied miRNAs (MiRNA-34a & miRNA-192) and other studied biochemical parameters were assessed using Person’s correlation. A *P*-value < 0.05 was deliberated statistically significant. The ROC curve was plotted, and the AUROC was estimated with a 95% confidence interval (CI) to recognize the diagnostic efficacy.

## Results

The present study is a case-control study consisting of 90 adult subjects (32 males and 58 females). Subjects were divided into two groups: the patient group included 60 NAFLD patients, and the control group included 30 healthy subjects. The patient group was further divided into two groups: the first group included 28 NAFLD patients at an early stage of fibrosis (F0-F2), and the second group included 32 NAFLD patients with advanced stage of fibrosis (F3-F4).

Table 1 provides an overview of the demographic, anthropometric, clinical, and biochemical data of both groups (NAFLD and controls). The average age of NAFLD patients was substantially higher than that of controls (45.22 ± 11.4 vs. 31.7 ± 10.7 years, respectively) (*P* < 0.001) (Table 1). Regarding gender distribution, there were more males in the control group (53.3%) compared to the NAFLD group (26.7%), but the majorities were females in the NAFLD group (73.3%). NAFLD patients displayed a higher mean BMI than controls. In the NAFLD and control groups, the mean BMI was 33.2 ± 7.18 and 23.99 ± 1.8 kg/m^2^, respectively (*P* < 0.001). The ANOVA analysis test revealed that the mean BMI of patients (with advanced fibrosis) increased significantly when compared to those with early fibrosis and the control group (*P* = 0.011 & *P* < 0.001; respectively) (Table 2).

**Table 1:** Characteristics of study participants in both studied groups

Variable	Total NAFLD group (***N*** = 60)	Control group (***N*** = 30)	***p***-value
Groups			
**Age (years)**	45.22 ± 11.4	31.7 ± 10.7	< 0.001**
**Gender**^a^			0.013*
**Male/female**	16/44	16/14	
**Percentage of male**	26.7%	53.3%	
**Hypertension**			0.012*
**No**	49 (81.7%)	30 (100%)	
**Yes**	11 (18.3%)	0 (0%)	
**Diabetes**			< 0.001**
**Absent**	29 (48.3%)	30 (100%)	
**Oral hypoglycemic**	18 (30%)	0 (0%)	
**On insulin**	13 (21.7%)	0 (0%)	
**BMI (kg/m**^**2**^**)**	33.2 ± 7.18	23.99 ± 1.8	< 0.001**
**Laboratory variables**			
**Fasting blood glucose (mg/dl)**	136.2 ± 76.8	93.2 ± 12.1	0.002**
**HB (g/dL)**	11.5 ± 1.3	12.7 ± 1.34	0.004**
**Platelets (10**^**3**^**/μL)**	224.1 ± 73.1	255.04 ± 58.3	0.110
**Total leucocytic count (10**^**3**^**/μL)**	6.47 ± 1.9	6.81 ± 1.7	0.518
**AST** (U/L)	25.7 ± 10.8	23.75 ± 5.8	0.279
**ALT** (U/L)	29.12 ± 17.9	28.5 ± 6.5	0.815
**Total bilirubin** (mg/dL)	0.66 ± 0.25	0.67 ± 0.2	0.947
**ALP** (U/L)	134.17 ± 50.01	115.46 ± 15.6	0.029*
**GGT** (U/L)	66.85 ± 49.5	37.46 ± 23.8	0.002**
**Total protein** (g/dL)	7.65 ± 0.48	7.88 ± 0.27	0.014*
**Serum albumin** (g/dL)	3.75 ± 0.4	3.8 ± 0.28	0.593
**Cholesterol** (mg/dl)	161.45±38	102.34 ± 24.14	< 0.001**
**Triglycerides** (mg/dl)	161.34 ± 89.07	145.6 ± 29.2	0.230
**HDL** (mg/dl)	51 ± 11.3	55.14 ± 12.3	0.128
**LDL** (mg/dl)	120.4 ± 31.2	100.1 ± 16.2	< 0.001**

*P < 0.05. **P < 0.01

**Table 2:** Clinical characteristics of cases with early fibrosis and advanced fibrosis as well as controls

Variable	Control group (***N*** = 30)	Early fibrosis (F0-F2) (***N*** = 28)	Advanced fibrosis (F3-F4) (***N*** = 32)	***p***-value
Groups				
**Age (yrs.)**	31.7 ± 10.7	43.21 ± 12.7^a^**	46.97 ± 10.74^a^**	< 0.001**
**Gender**^a^				0.032*
**Male/female**	16/14	9/19	7/25	
**Percentage of male**	53.3%	32.1%	21.9%	
**Hypertension**				0.043*
**No**	30 (100%)	23 (82.1%)	26 (81.3%)	
**Yes**	0 (0%)	5 (17.9%)	6 (18.8%)	
**Diabetes**				< 0.001**
**Absent**	30 (100%)	13 (46.4%)	16 (50%)	
**Oral hypoglycemic**	0 (0%)	7 (25%)	11 (34.4%)	
**On insulin**	0 (0%)	8 (28.6%)	5 (15.6%)	
**BMI (kg/m**^**2**^**)**	23.99 ± 1.8	31.11 ± 4.8^a^**	35.03 ± 8.4^a^**^,^^b^*	< 0.001**
**Laboratory variables**				
**Fasting blood glucose (mg/dl)**	93.24 ± 12.1	127.29 ± 63.1^a^*	149.87 ± 94.9^a^**	0.01**
**HB (g/dL)**	12.7 ± 1.34	11.4 ± 1.3 ^a^**	11.8 ± 0.6	0.015*
**Platelets (10**^**3**^**/μL)**	255.04 ± 58.3	234.53 ± 74	165 ± 27.2^a^*	0.064
**Total leucocytic count (10**^**3**^**/μL)**	6.81 ± 1.7	6.83 ± 1.8	4.39 ± 0.89^a,b^**	0.072
**AST** (U/L)	23.75 ± 5.8	23.87 ± 7.44	27.85 ± 13.7	0.189
**ALT** (U/L)	28.5 ± 6.5	35.9 ± 20.9	23.4 ± 12.4^b^**	0.006**
**Total bilirubin** (mg/dL)	0.67 ± 0.21	0.68 ± 0.3	0.64 ± 0.3	0.815
**ALP** (U/L)	115.46 ± 15.6	142.2 ± 45.4^a^*	120.2 ± 55.9	0.04*
**GGT** (U/L)	37.46 ± 23.8	75.08 ± 56.6^a^*	52.6 ± 30.3	0.005**
**Total protein** (g/dL)	7.88 ± 0.27	7.81 ± 0.43	7.37 ± 0.44^a,b^**	< 0.001**
**Serum albumin** (g/dL)	3.8 ± 0.28	3.74 ± 0.38	3.78 ± 0.44	0.833
**Cholesterol** (mg/dl)	102.34 ± 24.14	162.8 ±32.1^a^**	160.2 ± 43.5^a^**	< 0.001**
**Triglycerides** (mg/dl)	145.6 ± 29.2	166.5 ± 85.9	156.4 ± 93.2	0.586
**HDL** (mg/dl)	55.14 ± 12.3	53.7 ± 12.9	48.4 ± 8.9^a^*	0.071
**LDL** (mg/dl)	100.1 ± 16.2	127.9 ± 33.5^a^**	113.2 ± 27.6^b^*	0.001**

*P < 0.05. **P < 0.01. ^a^Significant difference with control group. ^b^Significant difference with NAFLD with early fibrosis group

In comparison to healthy controls, patients with NAFLD showed a higher prevalence of hypertension and diabetes mellitus (*P* < 0.05) (Table 1).

In terms of laboratory data, the findings demonstrated that NAFLD patients’ mean serum fasting blood glucose levels were substantially higher than those of controls ((136.2 ± 76.8 vs. 93.2 ± 12.1); (*P* = 0.002)).

Additionally, NAFLD cases had decreased hemoglobin levels (11.5 ± 1.3 (g/dL) compared to healthy controls (12.7 ± 1.34 (g/dL), with a *P*-value of 0.004.

Early fibrosis patients were found to have lower hemoglobin levels than healthy controls (11.4 ± 1.3 vs. 12.7 ± 1.34 (g/dL)) (*P* = 0.004) (Table 2).

However, no significant difference was observed in platelets count and total leucocytic count (TLC) between the NAFLD and control groups (*P* > 0.05).

There was no difference in both ALT and AST enzymes in NAFLD cases compared to control cases (*P* > 0.05). However, the ANOVA analysis test shows that there was a substantial drop in ALT levels in cases with advanced fibrosis compared to those with early fibrosis (23.4 ± 12.4 (U/L) vs. 35.9 ± 20.9 (U/L) (*P* = 0.006) (Table 2).

The patient’s other liver enzymes (ALP, GGT) were noticeably elevated than that in controls (*P* < 0.05). Also, total protein showed significant elevation in NAFLD cases compared to controls (*P* = 0.01). On the other hand, the mean albumin level was similar in the NAFLD group (3.75 ± 0.4 g/dL) to the control group (3.8 ± 0.28 g/dL).

Lipid profile parameters showed certain abnormalities as there was a significant elevation in total cholesterol and LDL cholesterol among NAFLD patients compared to controls, while there was a nonsignificant decrement in HDL in the NAFLD group as opposed to controls (*P* = 0.128) (Table 1). In addition, the difference in triglyceride between both studied groups was not significant (*P* = 0.230).

### Differentially expressed miRNA-34a and miRNA-192 using RT-qPCR

The present study showed that miRNA-34a is upregulated in NAFLD patients compared to controls, with a fold change of 2.22 ± 8, but without significant difference (*P* = 0.241) (Table 3).

**Table 3:** Fold expression of miRNA-34a and miRNA-192 in patients compared with those from healthy controls as determined by RT-qPCR

	CT	miR-16 CT (normalize)	***Δ***Ct	***ΔΔ***Ct	Mean fold change	***p***-value	Regulation
**miRNA-34a−**	**20.87 ± 3.49**	**16.04 ± 2.89**	**4.8 ± 4.34**	**3.57 ± 4.34**	**2.22 ± 8**	**0.241**	**Upregulated**
**miR-192**	**28.73 ± 3.3**	**16.2 ± 2.78**	**12.55 ± 4.14**	**−0.249 ± 4.14**	**11.46 ± 33.7**	**0.019**	**Upregulated**

When we divided NAFLD patients into patients at an early stage of fibrosis (F0-F2) and those at an advanced stage of fibrosis (F3-F4), interestingly, miRNA-34a is upregulated in NAFLD patients at the early stage of fibrosis compared to controls, with fold expression of 4.02 ± 11.49 (*P* = 0.05). However, miRNA-34a is downregulated in NAFLD patients at the advanced stage of fibrosis compared to controls, with fold expression of 0.65 ± 1.17 (*P* = 0.831) (Table 4).

**Table 4:** Fold expression of miRNA-34a in patients with early and advanced stage of fibrosis compared with those from healthy controls as determined by RT-qPCR

	**NAFLD cases with early fibrosis**						
**miRNA-34a**	**miRNA-34a** **CT**	**miR-16** **CT (normalize)**	***Δ*Ct**	***ΔΔ*Ct**	**Mean fold change**	***p*-value**	**Regulation**
	**21.81 ± 4.2**	**17.37 ± 3.16**	**4.44 ± 4.87**	**3.19 ± 4.87**	**4.02 ± 11.49**	0.05*	**Upregulated**
	**NAFLD cases with advanced fibrosis**						
	**miRNA-34a** **CT**	**miR-16** **CT (normalize)**	**Mean*Δ*Ct **	**Mean*ΔΔ*Ct**	**Mean fold change**	***p*-value**	**Regulation**
	**20.05 ± 2.59**	**14.89 ± 2.06**	**5.16 ± 3.87**	**3.9 ± 3.87**	**0.65 ± 1.17**	**0.831**	**Down**

*P<0.05

The results obtained indicate that this miRNA could potentially be used as a diagnostic biomarker in the early fibrosis stage of NAFLD.

Additionally, our results showed that miRNA-192 is upregulated in NAFLD patients compared to healthy controls 11.46 ± 33.7 (*P* = 0.019) (Table 3). It was detected that expression miRNA 192 in NAFLD cases at an early stage of fibrosis and those at an advanced stage of fibrosis have been increased compared to healthy controls with fold expression 18.43 ± 47.8 and 5.36 ± 8.89, respectively. ANOVA test results show that there is a significant difference in miRNA-192 levels between NAFLD patients at an early stage of fibrosis compared to the control group (*P* = 0.017). However, there is no significant difference in miRNA-192 level between NAFLD cases at the advanced stage of fibrosis compared to control (*P* = 0.530) (Table 5).

**Table 5:** Fold expression of miRNA-192 in patients with early and advanced stage of fibrosis compared with healthy controls as determined by RT-qPCR

	**NAFLD cases with early fibrosis**						
**miR-192**	**miR-192** **CT**	**miR-16** **CT (normalize)**	***Δ*Ct**	***ΔΔ*Ct**	**Mean fold change**	***p*-value**	**Regulation**
	**28.81 ± 1.99**	**17.67 ± 2.8**	** 11.1±2.59**	**−1.66 ± 2.59**	**18.43 ± 47.8**	0.017*	**Upregulated**
	**NAFLD cases with advanced fibrosis**						
	**miR-192** **CT**	**miR-16** **CT (normalize)**	**Mean** ***Δ*Ct**	**Mean** ***ΔΔ*Ct**	**Mean fold change**	***p*-value**	**Regulation**
	**28.67 ± 4.16**	**14.89 ± 2.06**	**13.78 ± 4.8**	**0.98 ± 4.8**	**5.36 ± 8.89**	**0.530**	**Upregulated**

*P<0.05

### Diagnostic efficacy of miRNA-34a and miRNA192

**Fig. 1:**
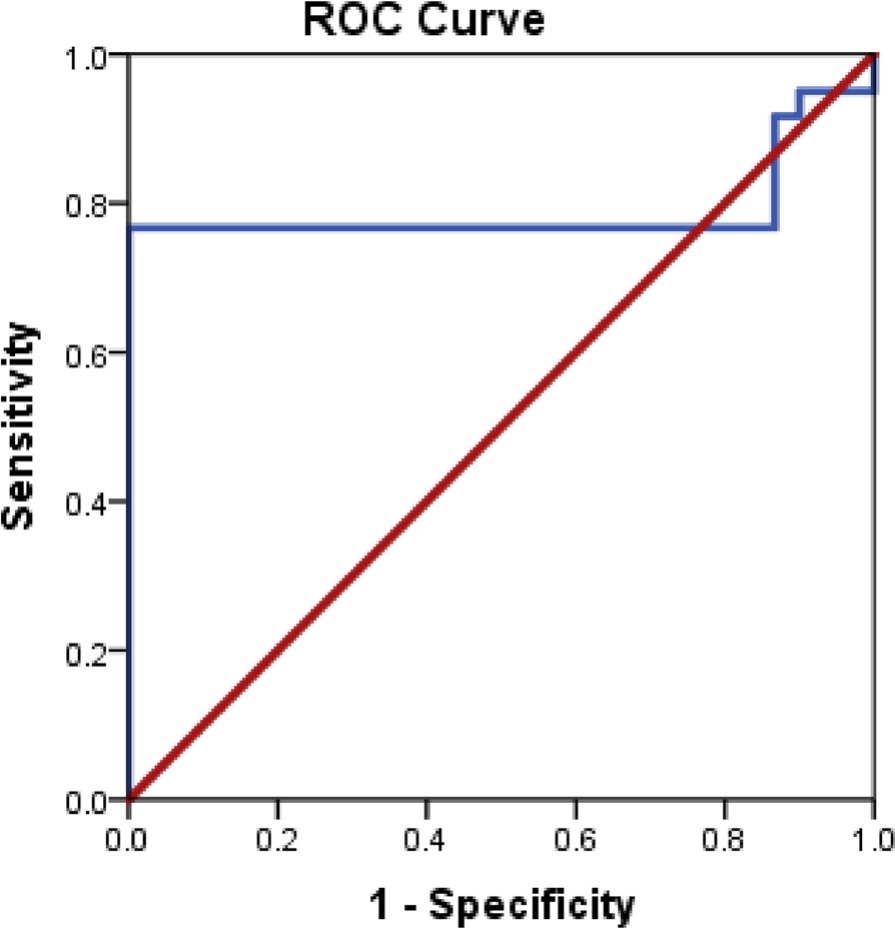
Nonparametric receiver operating characteristic (ROC) curves of miRNA-34a for distinguishing NAFLD patients from those healthy controls

**Fig. 2:**
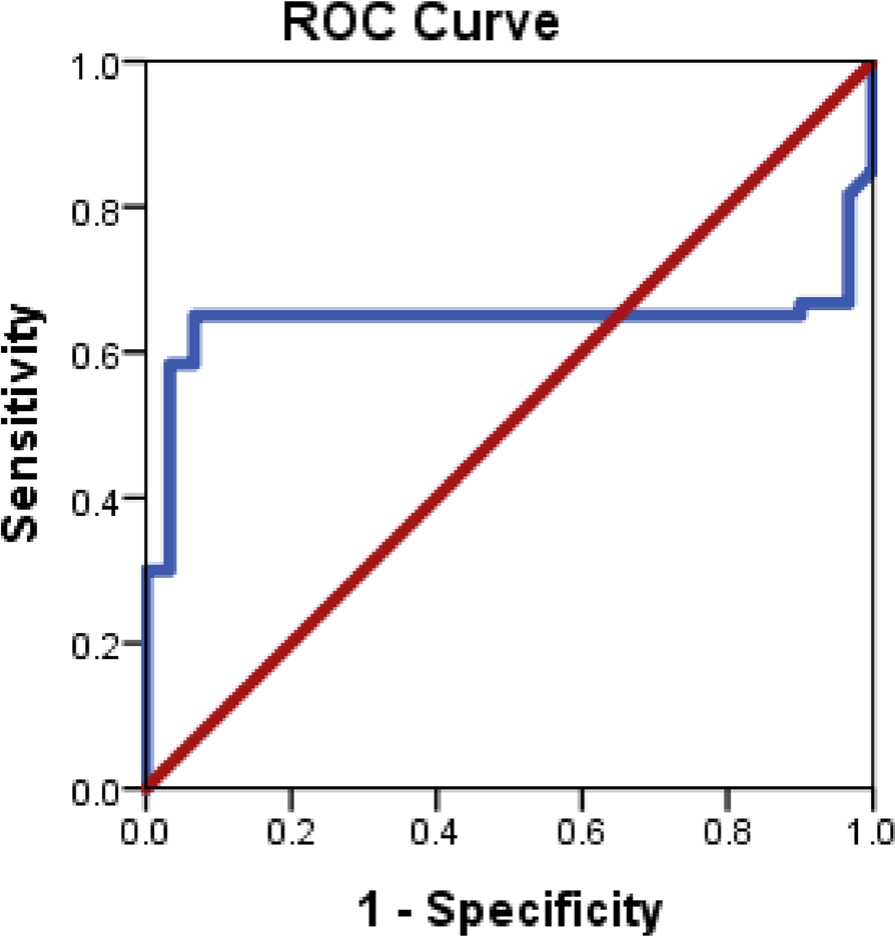
Nonparametric receiver operating characteristic (ROC) curves of miRNA-192 for distinguishing NAFLD patients from those healthy controls

Receiver operating characteristic curves were outlined to identify the diagnostic efficacy of the miRNA-34a and miRNA192 as shown in Figs. 1 and 2, the area under the curve for both miRNA-34a and miRNA-192 were (0.790 and 0.643, respectively), and the sensitivities and specificities were 76.7%, 100% for miRNA-34a and 63.3%, and 93.3% for miRNA-192, and both miRNAs reported to be significant (*P* <0 .05) (Figs. 1 and 2).

### Relation between investigated miRNAs and biochemical parameters

MiRNA34a reported a significant positive correlation with hypertension (*r* = 0.255, *P* = 0.037) and FBS (0.366, *P* = 0.016) and a significant negative correlation with hematological characteristics including hemoglobin level (*r* = −0.456, *P* = 0.022) and total leucocyte count (*r* = 0.417, *P* = 0.038).

Additionally, we found that the expression level of miRNA-34a− was also correlated with cholesterol level, but it did not reach a significant level (*r* = −0.233, *P* = 0.068).

However, there was no significant correlation between MiRNA-192 and any of the studied parameters (*P* > 0.05) (Table 6).

**Table 6:** Correlation of serum miRNA34a and miRNA192 with other studied parameters

	miRNA34a		miRNA192	
	***r***	***p***-value	***r***	***p***-value
**Age (yrs.)**	0.128	0.303	0.147	0.232
**Gender**^a^ **Male/female** **Percentage of male**	0.192	0.119	0.038	0.761
**Hypertension** **No** **Yes**	0.255	0.037*	0.075	0.541
**Diabetes** **Absent** **Oral hypoglycemic** **On insulin**	−0.005	0.967	0.000	1.000
**BMI (kg/m**^**2**^**)**	0.140	0.267	0.087	0.487
**Laboratory variables**				
**Fasting blood glucose (mg/dl)**	0.366	0.016*	0.177	0.251
**HB (g/dL)**	−0.456	0.022*	−0.194	0.342
**Platelets (10**^**3**^**/μL)**	0.043	0.839	−0.193	0.344
**Total leucocytic count (10**^**3**^**/μL)**	−0.417	0.038*	−0.335	0.095
**AST** (U/L)	−0.188	0.137	−0.217	0.083
**ALT** (U/L)	−0.123	0.332	−0.148	0.238
**Total bilirubin** (mg/dL)	**0.013**	**0.925**	**0.168**	**0.230**
**ALP** (U/L)	−**0.109**	**0.469**	**0.002**	**0.990**
**GGT** (U/L)	−**0.292**	0.049*	−**0.245**	**0.097**
**Total protein** (g/dL)	−**0.094**	**0.534**	−**0.273**	**0.064**
**Serum albumin (**g/dL)	−**0.061**	**0.691**	−**0.208**	**0.165**
**Cholesterol** (mg/dl)	**0.233**	**0.068**	**0.077**	**0.550**
**Triglycerides** (mg/dl)	**0.008**	**0.953**	**0.044**	**0.731**
**HDL** (mg/dl)	**0.095**	**0.464**	−**0.152**	**0.234**
**LDL** (mg/dl)	−**0.125**	**0.334**	−**0.090**	**0.482**
**miRNA-192**	**0.584**	< 0.001**	**1**	**-**

*P<0.05. **P<0.01

## Discussion

Currently, NAFLD is the most prevalent liver disorder resulting from excessive lipid buildup, liver cell destruction, and immune system malfunction that causes scarring and chronic liver damage that may eventually progress to HCC [23]. Early detection and diagnosis are necessary for clinical practice decision-making and may be beneficial in the management of patients with NAFLD. The present study aimed to investigate the significance of MiRNA-34a and MiRNA-192 as diagnostic biomarkers in NAFLD in the Egyptian population. The current study revealed a substantial correlation between nonalcoholic fatty liver disease progression and older age. The literature has already shown that the prevalence of NAFLD rises with age, and this data supports those findings [24]. In the current study, the prevalence of NAFLD has been rising along with the occurrence of other metabolic disorders such as dyslipidemia, diabetes, hypertension, and visceral obesity. As anticipated, patients with NAFLD had significantly increased rates of diabetes and hypertension. This is in line with earlier research that identified reduced glucose tolerance as a separate risk factor for the development of NAFLD [25, 26]. According to Lonardo et al., patients with T2DM had liver fat contents that were 80% higher than those of nondiabetic patients. Additionally, they noted that T2DM patients have a 2–4-fold greater risk of problems caused by the fatty liver in addition to an especially high risk of developing NASH [27].

The NAFLD group in the current study also had higher BMI than controls. This can be explained as obesity is associated with an increased risk of nonalcoholic fatty liver disease and hepatic steatosis. Our results are consistent with other research that found that NAFLD patients had significantly greater BMI and waist circumference (WC) than healthy controls. According to a recent survey, 30–100% of people with NAFLD are obese. Also, steatosis is 4.6-fold higher in obese cases than in normal-weight people [28, 29].

Except for hemoglobin levels, there was no significant correlation between nonalcoholic fatty liver disease and CBC parameters. Patients with NAFLD have considerably lower serum hemoglobin concentrations than healthy controls.

This is in agreement with El Nakeeb et al. who showed that hemoglobin level was lower in cases with simple steatosis compared to healthy controls, but without significant difference [30]. However, this is in contrast to prior studies which reported the role of hemoglobin in the development of NAFLD, but not steatohepatitis [31, 32].

Another study suggested that hemoglobin can protect against steatosis by acting as an antioxidant and reducing the disease’s negative effects, but this protective effect is lost when liver necrosis and inflammation occur [33].

The current study also demonstrated that NAFLD cases with early fibrosis had significantly higher levels of ALT than non-NAFLD individuals; however, this difference was not statistically significant. The elevation of ALT levels in NAFLD cases can be attributed to the role of this marker in necrosis and hepatocellular injury. Also, the raise of serum aminotransferases and elevated GGT imply oxidative stress and inflammatory activity in NAFLD [34–36]. However, the elevations in ALT and AST are usually mild and are usually not more than four times the upper limit of normal [37].

In NAFLD patients, the elevation in ALT usually surpasses the elevation of AST, and this is also observed in our study. This may be due to the high-calorie diet among Egyptians which lead to an increase in their transaminase levels owing to a greater influx of carbohydrates by glycolysis. Because ALT is directly engaged in pyruvate metabolism, its elevation outpaces that of AST [38, 39]

Additionally, the current study is consistent with prior studies which showed an association between elevated serum total cholesterol, LDL, and triglyceride levels, as well as reduced HDL levels and the presence of NAFLD [40, 41]. This could be due to the hepatic overproduction of very low-density lipoprotein (VLDL) particles and dysregulation in the clearance of various lipoproteins from the circulation [42].

The two miRNAs that have been studied the most in NAFLD patients to date are miRNA-34a and miRNA-192. As a tumor suppressor miRNA, miRNA-34a may have a key role in hepatocellular carcinogenesis, as was previously mentioned. According to previous reports, different classes of cancers’ growth and development are reportedly aided by aberrant miRNA-34a expression [43]. Additionally, miRNA-34a is known to assist in controlling lipid and cholesterol metabolism, inflammation, and other processes in mouse or human liver tissues [44]. In mice given a high-fat diet, miRNA-34a expression demonstrated a significant association with NAFLD susceptibility [45]. It was detected that antisense suppression of miRNA-34a was found to improve metabolic gene expression and metabolic outcomes in obese mice [46].

MiRNA-34a expression was found to be higher in NAFLD patients compared to controls but without significant differences. Our finding, however, revealed that miRNA-34a could only distinguish NAFLD patients with early fibrosis from healthy controls. Our results are consistent with a recent study that profiled several miRNAs expression in NAFLD [19].

According to the study, miRNA-34a could be used to distinguish between individuals with NAFL from healthy controls. However, this study also reported that miRNA-34a could be used to distinguish between individuals with NASH and healthy controls.

Additionally, other studies reported the increment of miRNA-34a levels in the serum and liver of NAFLD patients and animal models [10, 14, 20].

However, Salvozaet al. (2016) noted that there was no association between serum miRNA-34a relative expression and the advanced stage of fibrosis [44]. Variability in RNA isolation and detection methods could be one explanation for these contradicted results. Also, using MiRNA-16 to normalize the gene expression of both studied miRNAs could be another explanation as until now; there is no consensus on the use of housekeeping microRNAs for qRT-PCR.

Our research revealed a strong association between miRNA-34a and NAFLD risk factors such as hypertension and FBS. We also discovered a correlation between cholesterol levels and miRNA-34a expression; however, it was not statistically significant. In accordance with other studies, it was detected that miRNA-34a promotes liver steatosis and hypolipidemia while regulating lipoprotein metabolism in a PPAR-dependent manner [17].

As opposed to miRNA-34a, miRNA-192 has been discovered as an oncogene in several malignancies, including colon, breast, and gastric cancers [21]. It is also adversely correlated with the ability of colon cancer cells to spread to the liver [47].

Our results showed that miRNA-192 is upregulated in NAFLD patients compared to healthy controls (*P* = 0.019). It was detected that NAFLD cases at an early stage of fibrosis but not those at the advanced stage of fibrosis have a significant increase in miRNA-192 expression compared to healthy controls. This is in agreement with previously published studies that reported increased serum miRNA-192 levels in NAFLD [9, 12]. Due to its role in lipid synthesis through targeting stearoyl-CoA desaturase 1, miRNA-192 stands out among the other miRNAs associated with NAFLD; consequently, its overexpression would be a strategy to treat the condition [48, 49].

In addition, it has been noted that miRNA-192 expression is decreased in the liver of NASH patients compared to NAFLD patients [10, 12, 19]. However, this is in contrast with Kim’s study which mentioned that miRNA-192 was consistently upregulated in NASH vs. NAFL [50]. Exosomes from lipotoxic hepatocytes displayed elevated miRNA-192-5p, as exosomal miRNA-192-5p adjusts NAFLD progression via stimulating proinflammatory macrophages

## Conclusion

NAFLD is associated with changes in hepatic miRNA expression patterns at early, intermediate, and late stages, and specific miRNA species appear to be involved in steatosis development and NAFL progression to NASH and cirrhosis. Our study demonstrated that the serum levels of both miRNA-34a and miRNA-192 are upregulated in NAFLD patients compared to healthy controls and could be considered as best candidates for NAFLD diagnosis and staging. Additionally, both studied miRNAs showed significant association with the early stage of fibrosis so they can be used to predict the occurrence of NAFLD at an early stage.

Furthermore, our research revealed a strong association between miRNA-34a and NAFLD risk factors such as hypertension and fasting blood sugar which indicated that miRNA-34a promotes liver steatosis and may play a key role in the onset of the disease. Further studies are required to confer the diagnostic and prognostic value of both studied miRNAs in the detection of NAFLD and to elucidate the mechanism of these biomarkers in the progression of disease.

## Data Availability

The datasets used and/or analyzed during the current study are available from the corresponding author on reasonable request.
